# A comparative study of machine learning algorithms for predicting domestic violence vulnerability in Liberian women

**DOI:** 10.1186/s12905-023-02701-9

**Published:** 2023-10-17

**Authors:** Riaz Rahman, Md. Nafiul Alam Khan, Sabiha Shirin Sara, Md. Asikur Rahman, Zahidul Islam Khan

**Affiliations:** Statistic discipline, Khulna University, Khulna, 9208 Bangladesh

**Keywords:** XGBoost, Decision tree, K-NN, CatBoost, Domestic Violence, Machine learning technique, Prediction; Liberia, DHS

## Abstract

Domestic violence against women is a prevalent in Liberia, with nearly half of women reporting physical violence. However, research on the biosocial factors contributing to this issue remains limited. This study aims to predict women’s vulnerability to domestic violence using a machine learning approach, leveraging data from the Liberian Demographic and Health Survey (LDHS) conducted in 2019–2020.

We employed seven machine learning algorithms to achieve this goal, including ANN, KNN, RF, DT, XGBoost, LightGBM, and CatBoost. Our analysis revealed that the LightGBM and RF models achieved the highest accuracy in predicting women’s vulnerability to domestic violence in Liberia, with 81% and 82% accuracy rates, respectively. One of the key features identified across multiple algorithms was the number of people who had experienced emotional violence.

These findings offer important insights into the underlying characteristics and risk factors associated with domestic violence against women in Liberia. By utilizing machine learning techniques, we can better predict and understand this complex issue, ultimately contributing to the development of more effective prevention and intervention strategies.

## Highlights

Chi-square association was used to determine the significant factors for Domestic violence (DV) in Liberia by using LDHS 2019–20 data.

LightGBM and RF performed the best of all seven machine learning models, with the highest accuracy and F1 score.

Victims of emotional violence were the most important feature of almost every model.

## Introduction

Domestic violence, also known as Intimate partner violence (IPV), is the threat or use of physical, psychological, or emotional abuse towards a partner [1]. It is frequently manifested by gender-specific violence driven by sexism and gender inequity [2].Domestic violence is simply characterized as a man’s assault on a woman [3]. Women are experiencing unprecedented levels of violence in modern society. It is widely acknowledged as a serious violation of human rights and a global health concern [4].

Domestic violence is being put into a global context through things like feminist theories of violence, studies of cyberviolence, and works on privacy. Several researches suggest how a culture where males predominate causes violence against women and restricts women’s bodies in public areas, sexual expression, overall appearance, careers, and spare time. Cyberviolence against women also includes threats of rape, the sharing of private information, and the spreading of photos of a noose that have been changed. Also, domestic violence is linked to not having enough privacy at home [5]. The rates of domestic violence have been increased during the COVID-19 [6]. WHO research shows that the rate of violence between people who live together varies a lot from country to country, from 15% in Japan to 71% in rural Ethiopia [7]. Lifetime rates of emotional domestic abuse in Ethiopia were found to be 51.7%, while rates of physical and sexual assault were found to be between 19.2 and 59.0% [4]. According to a recent global study, businesses can have a significant impact on reducing domestic violence. Nine per cent or more of workers have experienced sexual harassment on the job.

Nevertheless, among all forms of domestic violence, emotional abuse was by far the most prevalent [8]. Characteristics such as maternal education, housing, an unplanned pregnancy, partner substance usage, and a lack of prenatal care were associated with a higher risk of experiencing domestic violence [9]. Not only that, but being a housewife, having fewer children, marrying at a younger age, having a shorter marriage, and having a husband who is unemployed are all connected [8]. In a Pakistani study, poverty, in-law influence, second marriage, stepchildren, forced personal connections, the husband’s irresponsibility and drug use, and children with disabilities were all named as risk factors for domestic violence [10, 11]. Victims of intimate partner abuse had worse mental health, including depression, PTSD, and anxiety, than non-victims [12]. Undesirable physical and mental health effects, especially traumatic brain damage, chronic pain, insomnia, pelvic pain, depression, and post-traumatic stress disorder, may arise from IPV [13–16].

The high prevalence of sexual and other forms of gender-based violence in armed conflict was once considered an unfortunate but unavoidable side effect of war [17]. Violence and rape are hard to prove everywhere, but especially in Liberia, which has been in conflict from 1989 to 2003 and has seen damage to its infrastructure and social fabric [18, 19]. Two hundred seventy thousand people died from violence or illnesses that went untreated because they couldn’t get to a hospital or get medicine. Over700,000 people escaped Liberia, and more than 1.4 million were internally displaced [20]. In Montserrado and Nimba, more than half of women had been hurt by their partners in ways that were not sexual, and 20% had been raped outside of marriage [20].Nearly half (45%) of individuals in post-conflict Liberia reported physical violence from non-partners [21]. Domestic violence has disempowered women in West Point, Liberia by causing low self-esteem, dependence, low skills, low self-confidence, trauma, stigma, and job loss [22]. Intimate partner violence has a devastating effect on women’s health, both psychologically and physiologically [23]. Furthermore, women’s independence in Liberia has been hampered by domestic abuse. Home abuse adversely affects children’s cognitive growth and mothers’ mental and emotional well-being. Many initiatives have been launched to guarantee women’s safety and promote their independence. There is a lack of quality biosocial studies examining the topic of domestic violence in Liberia.

This study aims to present the recent prevalence of domestic violence, the associated factors, and also to predict domestic violence using machine learning. The ANN, KNN, RF, DT, XGBoost, LightGBM, and CatBoost algorithms.

However, there has been no investigation of domestic violence in Liberia utilizing ANN, KNN, RF, DT, XGBoost, LightGBM, and CatBoost algorithms with the most current data from the Liberian Demographic and Health Survey (LDHS). We will focus on using machine learning algorithms rather than deep learning because of the structured and limited datasets, and also to avoid overfitting. In our study, we will run six different machine learning (ML) algorithms and compare how well they can predict things to find the best predictive model for our study. This study differs from others in the way features are selected and the accuracy of 10-fold cross-validation. The classifiers will also help policymakers find features of domestic violence early on so they can stop it and help Liberian women and future generations have a better world.

## Review of related works

There are few related works of domestic violence in Liberia, but we found some recent research who used machine learning algorithms in similar work. A research evaluated the high-effect factors of citizens’ happiness, but it had constraints regarding data, algorithm selection, processing overhead, and real-time detection. The dataset utilized is undiversified, which makes machine learning models challenging to generalize. Furthermore, the algorithms used (SVM-RBF and IB-KNN) may not be optimal, and other methods should be investigated. Additionally, ethical concerns like censorship and privacy in automated systems must be addressed. Finally, practicality and effectiveness must be assessed through real-world installation and evaluation in email servers, social media platforms, and news websites [24].

Another study used machine learning algorithms to detect unipolar and bipolar depression detection on actigraphic registration of motor activity. However, the dataset’s uneven class distribution needs to improve the analysis. This imbalance, particularly in Bipolar II patients, might distort results and impair the model’s real-world applicability. Strategies such as oversampling or synthetic data production should be investigated to solve this issue. Furthermore, there is no investigation of the interpretability and explainability of depression categorization models in the study. Understanding how these models make predictions is critical, especially when trust and transparency are essential in a therapeutic setting [25]. To address these limitations and advance the field of machine learning in depression detection, future research should consider strategies for class imbalance mitigation, more extensive and diverse datasets, improved model interpretability, validation in clinical settings, and comparison of dimensionality reduction techniques.

In 2022, a study suggested a directed network link prediction approach based on path extension similarity to increase the forecast accuracy of network node potential edges. The study mentions the use of numerous real data sets for accuracy verification and robustness analysis. However, how far the proposed strategy may be applied to different networks or datasets is still being determined. ML researchers frequently look for approaches that generalize well across domains and datasets. The research focuses on link prediction in directed networks using a particular approach (path extension similarity). The proposed method may only be applied to some sorts of ML issues, which is a limitation [26].

A researched proposed novel ensemble and robust anomaly detection method based on collaborative representation-based detector. The focused pixels utilized to estimate the background data are drawn randomly from the image. A critical disadvantage in this study is the unpredictability induced by using randomly picked pixels to estimate background data. Furthermore, while the publication claims that the approach is less sensitive to outliers, a thorough assessment of its robustness is required. This should include assessing its performance in various outlier scenarios involving various types and degrees of anomaly to better understand its strengths and limitations [27].

A study on influencer marketing sheds light on the influence of fake followers on perceptions of influencing power. However, it has numerous shortcomings that highlight topics for further research in machine learning (ML) applications in influencer marketing. While the study analyzes the impact of false followers, it does not directly investigate the application of machine-learning approaches to detect or quantify fraudulent followers. Given the constantly developing strategies employed by influencers to disguise themselves, future studies could dive into the creation and implementation of ML models for more precise and automatic identification of false followers [28].

An article that used the multi-modal fusion in visual question answering highlights the role of attention mechanisms in Visual Question Answering (VQA) but does not detail ML testing for these mechanisms. Future research should build standardized testing procedures and datasets for evaluating attention-based models in cross-modal retrieval scenarios to ensure their dependability in practical applications [29]. An article in 2023 recently used an improved multi-label method to classify emotions for short texts. The study focuses on improving classification accuracy and speed but needs to extensively address the challenges related to noisy or ambiguous data in real-world Twitter conversations. The study’s experiments were also conducted on a Twitter corpus, which may not fully represent the diversity of short text data found on other social media platforms or digital environments [29].

A research combined the multi-layer semantic representation network with the deep fusion matching network to overcome the restrictions of merely examining a sentence representation module or a reasoning model. It does not, however, provide explicit solutions for dealing with the mutual limitations identified between these modules. While the joint optimization model improves recognition accuracy, the paper notes there is still potential for improvement. ML testing should investigate sophisticated optimization strategies and novel model architectures to obtain even higher reasoning accuracy, potentially pushing the bounds of natural language reasoning capabilities. [30]. In 2023, a study recognized extended dialogue emotion using commonsense knowledge graph guidance; however, the work relies on external commonsense knowledge from the ATOMIC atlas and does not examine potential limits or biases in this knowledge source. External knowledge’s correctness and completeness can impact emotion detection quality; therefore, any limits in the ATOMIC dataset should be considered [31].In our research, we embark on a comprehensive exploration of machine learning (ML) algorithms, employing six distinct models to discern their efficacy in predictive modeling. Our objective is to determine the most proficient predictive model for our study, which focuses on forecasting domestic violence among Liberian women. To achieve this, we harness the power of Artificial Neural Networks (ANN), K-Nearest Neighbors (KNN), Random Forest (RF), Decision Trees (DT), XGBoost, LightGBM, and CatBoost algorithms.

It is worth noting that, to the best of our knowledge, there has been a paucity of research endeavors that have utilized these advanced ML models for predicting domestic violence. This study endeavors to contribute novelty to this field by applying cutting-edge AI models to address critical women’s health issues, particularly in the context of domestic violence within underdeveloped countries.

Our study distinguishes itself from prior research in two pivotal aspects. Firstly, we employ a unique approach to feature selection, offering a novel perspective on identifying crucial indicators of domestic violence. Secondly, we rigorously assess our models’ performance through a rigorous 10-fold cross-validation process, enhancing the reliability and robustness of our findings.

The outcomes of our research hold significant potential for policymakers and advocacy groups. By identifying early indicators of domestic violence, our classifiers can play a pivotal role in proactively addressing and mitigating this pressing issue. Ultimately, our work aspires to contribute to a brighter future for Liberian women and subsequent generations, fostering a safer and more equitable world.

## Materials and methods

### Data collection

The investigation utilized data from the 2019-20 Liberia Demographic and Health Survey (LDHS) [32], which was conducted by the Liberia Institute of Statistics and Geo-Information Services (LISGIS) with permission from the Ministry of Health (MOH). This survey is the fifth in a series of demographic and health surveys conducted in Liberia.

### Study design and settings

The 2019-20 LDHS sample frame was created based on the Liberia Institute of Statistics and Geo-Information Services (LISGIS)’s 2008 NPHC. Liberia is divided into 15 counties, into five zones with three counties each, and further organized into clans. Each clan was partitioned into enumeration areas (EAs) during the 2008 NPHC. The census frame shows that each EA has an average of 100 households.

The LDHS 2019-20 utilized a two-stage cluster design. First, EA sample sites or clusters were selected. The EAs within each sample stratum were selected based on their size, resulting in a total of 325 cluster groupings. Second, households were selected from each cluster. The households were listed during the listing, and on average, 129 households were located in each cluster. From the listed households, 30 were randomly selected, resulting in a total sample size of 9,745 households. This sample is representative at the national, urban, and rural levels and covers all five regions, including the 15 counties [33]. After excluding type errors, missing, and unnecessary values, the remaining sample size was 1,907, which provides representative data for important metrics in all 15 counties.

### Measures

#### (1) Dependent variable

Ever having witnessed domestic violence was the dependent variable in our study. Two categories, “Yes” (experienced domestic abuse) and “No” (did not experience domestic violence), are used to classify the dependent variable. One indicates “Yes”, whereas zero indicates “No”.

#### (2) Independent variable

As an independent variable, we used the sociodemographic information of the respondents. Region (North Central, North Western, South Central, South Eastern A, and South Eastern B), Residence (Urban, Rural), Educational Level (No Education, Primary, Secondary, Higher), Body Mass Index (BMI), Wealth Index (Poor, Middle, Rich), Religion (Christian, Muslim, Traditional Religion, No Religion), Partner’s Education Level, Partner’s Occupation (Did not work, Public or private sector, Sales & services, Agricultural Sector, Skilled & Unskilled Manual), Respondent’s Occupation, Partner’s Age (11–20, 21–30, 31–40, 41–50, 51–60, 61–70, > 71), Lack of Independence (Yes, No), Victims of Emotional Violence (Yes, No), and Partner’s drinking habit (Yes, No) were among the sociodemographic factors. To classify BMI, the BMI standard scale was applied [34].

### Data splitting and model building

We used 80% of the dataset for training and 20% for testing. Using the training data, we fitted a variety of models. To ensure robustness, we tuned the model to find the ideal hyperparameters using grid search and 10-fold cross-validation. After creating the ultimate best model, the fitting process was repeated on the training set. Finally, we evaluated the model’s performance using an independent test set, which gave us helpful information on the predictability of our method for classifying domestic violence.

### Workflow chart for predicting DV

**Fig. 1:**
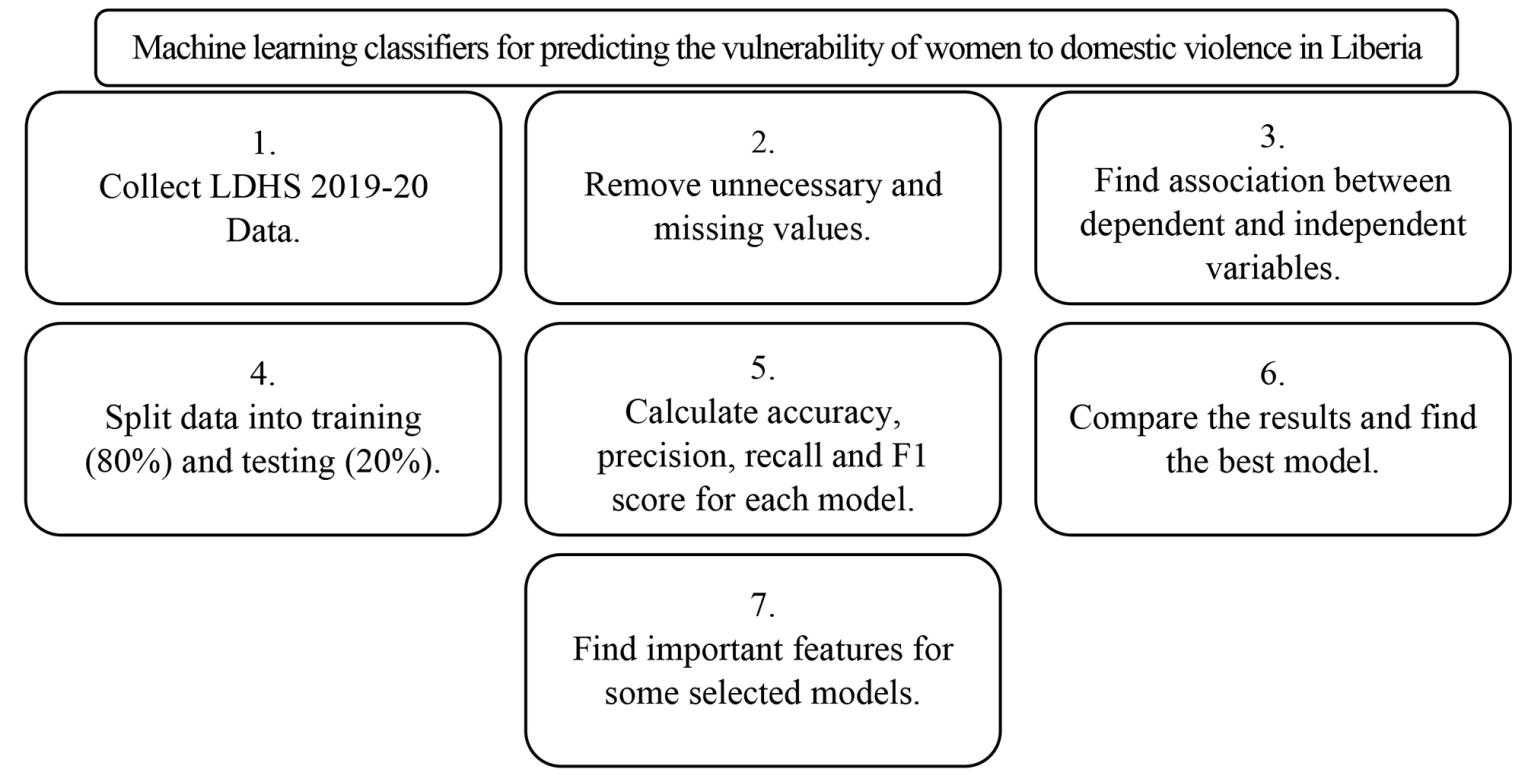
Workflow chart of domestic violence vulnerability in Liberian women

Figure 1 shows the workflow of the Machine learning classifiers for predicting the vulnerability of women to domestic violence in Liberia.

### Predictive model generation

We have applied seven distinct machine learning models to assess the accuracy of each model for our dataset to forecast the incidence of domestic violence in Liberia. Only some studies generate their methodology using machine learning models [35–38]. Brief descriptions of the following models are provided below:

### Artificial neural network (ANN)

The phrase “artificial neural network” refers to a subfield of artificial intelligence influenced by biology and fashioned after the brain. Typically, an artificial neural network is a computer network modeled after the biological neural networks that comprise the structure of the human brain. Similar to how neurons in the real brain are interconnected, artificial neural networks likewise contain interconnected neurons that multiple network levels. These neurons are referred to as nodes [38]. ANNs are used in various applications, including image and speech recognition, natural language processing, and predictive analytics.

Another criticalaspect of ANN model is its architecture, which includes the number of layers, the number of neurons per layer, and the activation functions used. In an ANN, the input is sent from the neurons in one layer to the neurons in the next layer after the bias and weight vectors have modified it. In the neurons of the hidden layer, information is processed, and the signal is modified using an activation function before being transmitted to the outer layer. Table 1 shows the hyperparameter values of the ANN model.

**Table 1:** Control parameters for ANN model for predicting DV

Parameters	Values
Random state	90
Input Dimension	12
Activation	relu
Metrics	accuracy
Optimizer	adam

### K-nearest neighbor (KNN)

K-nearest neighbor is a machine learning approach considered straightforward [39]. A categorization system based on similarities can be used to map the prediction of the domestic violence problem. A collection of vectors is created by mapping the test data and the historical observed data. The N-dimensional representation of each vector correlates to a feature. For classification tasks, KNN takes the majority class among the K closest neighbors as the predicted class for the new data point. For regression tasks, KNN takes the average (or weighted average) of the K closest neighbors as the predicted value for the new data point. Then, a similarity measure such as Euclidean distance is calculated to make a choice. A description of KNN is given in this section. When using KNN, which is regarded as lazy learning, no prior model or function is built; instead, the closest K records from the training data set that are most similar to the test are produced (i.e., query records). The class label is then decided by a majority vote among the chosen K records, and it is subsequently applied to the query record.

The following is how the prediction of domestic violence is calculated using KNN:

1. We calculated the K closest neighbors.
2. Calculated distance between the query record and the training samples.
3. Organize all training records by distance values.
4. Assign the prediction value for the query record to the class labels of the K neighbors with the most votes.

Table 2 represents the values of parameters for developing KNN model.

**Table 2:** Control parameters for KNN model for predicting DV

Parameters	Values
Random state	0
Number of neighbors	50
P	1
Weights	uniform
Validation type	K-fold
Number of validations	10

### Random Forest (RF)

Random Forest (RF) is a machine learning algorithm that belongs to the ensemble methods family. Ensemble methods combine multiple base models to improve the overall performance of the prediction task. Random Forest is a supervised learning algorithm that can be used for both regression and classification problems. American academic Leo Breiman of the University of California, Berkeley initially devised the random forest algorithm in 2001. It uses supervised data mining techniques. It uses supervised data mining techniques [40].

Table 3 shows the parameter values of RF model.

**Table 3:** Control parameters for RF model for predicting DV

Parameters	Status
Random state	42
Criterion	gini
Maximum depth	10
Number of estimators	100
Validation type	K-fold
Number of validations	10

### Decision tree (DT)

A well-liked supervised machine learning technique called Decision Tree (DT) is utilized for both classification and regression problems. The primary purpose of a DT is to build a tree-like model of decisions and potential outcomes using a collection of input features. Recursively dividing the data into progressively smaller sections depending on the values of the input characteristics creates the tree. A decision tree is a grid or tool used to enable choices among several alternatives, such as event outcomes, resource use, costs, and their application [41]. It is one method of displaying an algorithm. Decision tree applications are frequently utilized in decision research and analysis. Here we used Decision tree Classification. In general, it is employed for prediction purposes. It painstakingly analyses the data contained in vast facts to find useful patterns and relationships. In this work, a decision tree classifier from the Scikit-learn Python package is used [42].

The control parameter values of DT model are shown in Table 4.

**Table 4:** Parameters for DT model

Parameters	Attributes
Criterion	gini
Maximum depth	2
Minimum samples leaf	0.12
Validation type	K-fold
Number of validations	10

### Xtreme gradient boosting (XGBoost)

It is an implementation of gradient boosting, an ensemble learning method combining multiple weak models to form a robust model. The ensemble machine learning method XGBoost (Extreme Gradient Boosting) uses a gradient boosting framework, which is decision tree based. Only the first derivative information is used in conventional boosting tree models. The residual of the previous n-1 trees is employed while training the nth tree, making dispersed training challenging. XGBoost expands the loss function in a second-order Taylor manner and can automatically utilize the CPU’s multithreading for parallel processing. Additionally, XGBoost employs a number of techniques to prevent overfitting [43].

Some of the key features of XGBoost include:

1. Regularization: To avoid overfitting, XGBoost offers a number of regularisation approaches, including L1 and L2 regularisation.
2. Cross-validation: To identify the ideal number of boosting rounds, XGBoost includes cross-validation built in.
3. Handling missing values: The dataset’s missing values can be handled by XGBoost since it automatically learns how to manage them during training.
4. Processing in parallel: XGBoost is incredibly quick and scalable because it can parallelize the calculation of trees during training.

Table 5 shows the parameter values of XGBoost model.

**Table 5:** Control parameters for XGBoost model for predicting DV

Parameters	Values
Booster	gbtree
Colsample bylevel	1
Colsample bynode	1
Colsample bytree	0.3
Gamma	0.3
Grow policy	depthwise
Learning rate	0.05
Maximum bin	256
Maximum depth	8
Minimum child weight	7
Number of estimators	100
Random state	0
Regression alpha	0
Regression lambda	1

### Light gradient boosting (LightGBM)

**Fig. 2:**
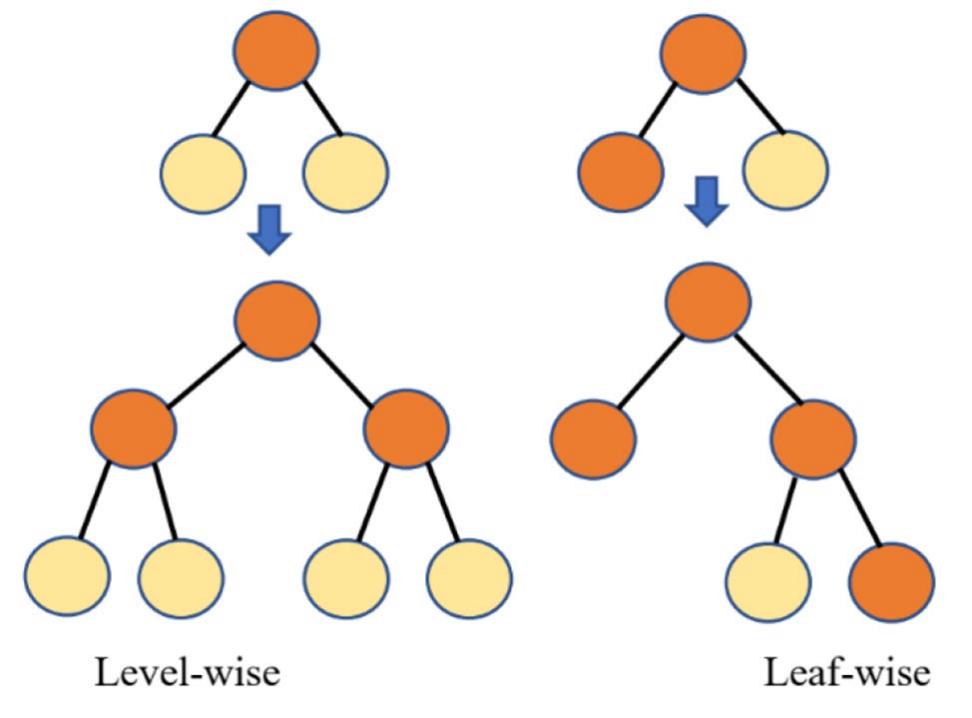
The tree generating technique in LightGBM [44]

Microsoft created the open-source GBDT (gradient boosting decision tree) algorithm, LightGBM. The parallel voting decision tree approach employs the histogram-based technique to speed training, reduce memory usage, and combine advanced network connectivity to maximize parallel learning. Each iteration involves splitting the training data into different machines, doing local voting to choose the top k attributes, and global voting to receive the top 2k attributes. To identify the leaf with the most significant splitter gain, LightGBM employs a leaf-wise approach (see Fig. 2). Because of its precision, effectiveness, and stability, GBDT is now frequently employed [44].

Key characteristics of LightGBM include:

1. Faster training speed: Training is completed more quickly, it can handle big datasets with millions of instances and characteristics.
2. Less memory usage: LightGBM employs a cutting-edge method called Gradient-based One-Side Sampling (GOSS) to save memory.
3. More precision: LightGBM employs exclusive feature bundling (EFB) to increase accuracy. EFB aggregates characteristics with comparable values into a single feature.
4. Tunable parameters: Varioushyperparameters, including the learning rate, the number of leaves, and the maximum depth, are offered by LightGBM and may be adjusted to enhance performance.

Table 6 shows the values of control parameter of LightGBM model.

**Table 6:** Parameters for LightGBM model

Parameters	Status
Colsample bytree	0.6847074798584072
Minimum child samples	125
Minimum child weight	10
Number of estimators	5000
n_jobs	4
Number of leaves	49
Random state	314
Regression alpha	5
Regression lambda	50
Subsample	0.20892704149103666

### Categorical boosting (CatBoost)

One of the most recent boosting algorithms is CatBoost (Categorical Boosting). Although it functions similarly to the Gradient and XGboost methods and is also intended to work with categorical information, this approach has advanced features that make it more dependable, quick, and accurate. The following are the benefits of CatBoost over other GBDT algorithms:

First off, categorical features are well handled by this method. With the appropriate average label value, categorical characteristics can be replaced by the conventional GBDT algorithm. The decision tree’s average label value will serve as the node-splitting criterion. CatBoost, in addition, incorporates various category traits. All categorical characteristics and combinations in the current tree are combined with all categorical features in the dataset using a greedy method by CatBoost. Thirdly, the gradient bias can be eliminated with CatBoost. In GBDT, a weak learner is generated in each iteration, and each learner is trained using the gradient of the previous learner. The output is the sum of the classified results from all of the learners [45].

The control parameter values of CatBoost model shown in Table 7.

**Table 7:** Parameters for CatBoost model for predicting DV

Parameters	Status
Iterations	15,000
Loss function	Logloss
Depth	8
Evaluation metric	AUC
Learning rate	0.03
Border count	32
Validation type	K-fold
Number of validations	10

## Results

**Fig. 3:**
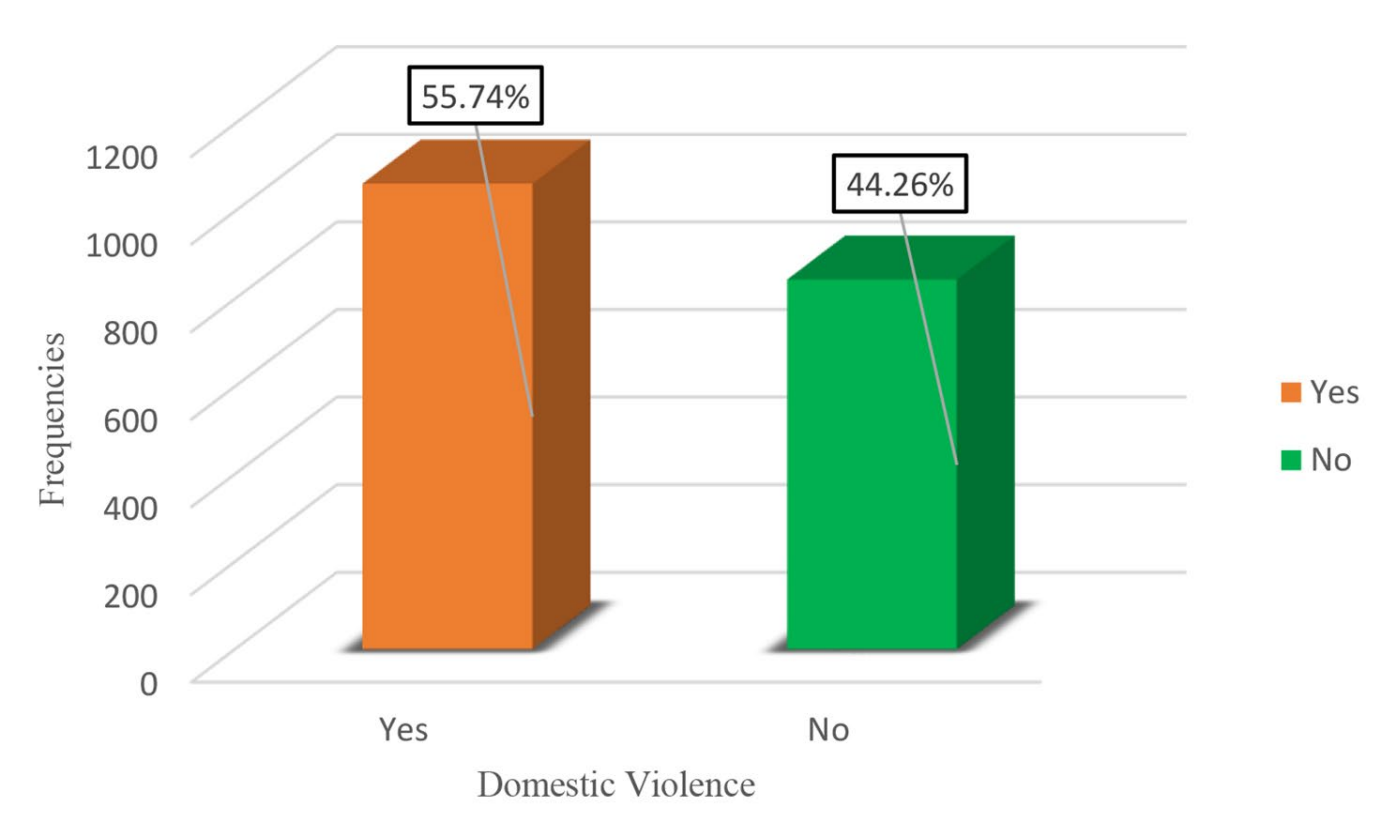
Bar chart of dependent variables (Domestic Violence)

The dependent variable was shown as a bar chart in Fig. 3, which showed that 55.74% of the participants had experienced domestic violence while 44.26% had not.

Table 8 shows how often domestic violence happens and the selected covariates’ background characteristics. Women in the South-Eastern B region had a greater likelihood of experiencing domestic abuse (51.4%) than women in other regions. Women had a higher risk of experiencing domestic violence if they came from a Christian family (44.2%), lived in a rural area (44.5%), received only primary education (50.2%), belonged to a rich family (45.7%), or if their husband or partner had only primary education (47.8%). Women between the ages of 21 and 30 also had a higher risk of experiencing domestic violence (52.2%). 48.0% of women who worked in sales and services; 51.1% of women who lacked independence; 78.1% of women who experienced emotional violence; and 78.3% of women whose husbands or partners had a drinking problem were at increased risk (59.0%). It was revealed that there was a significant link between the chosen covariates and the incidence of domestic violence against women (P < 0.001).

**Table 8:** Association between selected features with domestic violence in Liberia

Features	Categories	Domestic violence			${\varvec{\chi }}^{2}$	P-value
		No (%)	Yes (%)	Total (%)		
Region	North Western	166 (54.1)	141 (45.9)	307 (16.1)	11.43	0.022
	South Central	261 (58.8)	183 (41.2)	444 (23.3)		
	South Eastern A	200 (59.5)	136 (40.5)	336 (17.6)		
	South Eastern B	171 (48.6)	171 (51.4)	352 (18.5)		
	North Central	265 (56.6)	203 (43.4)	468 (24.5)		
Residence	Urban	341 (56.3)	265 (43.7)	606 (31.8)	0.10	0.751
	Rural	722 (55.5)	579 (44.5)	1301 (68.2)		
Educational level	No education	557 (59.8)	374 (40.2)	931 (48.8)	17.58	0.001
	Primary	256 (49.8)	258 (50.2)	514 (27.0)		
	Secondary	224 (52.8)	200 (47.2)	424 (22.2)		
	Higher	26 (68.4)	12 (31.6)	38 (2.0)		
Religion	Christian	917 (55.8)	727 (44.2)	1644 (86.2)	7.29	0.063
	Muslim	135 (57.9)	98 (42.1)	233 (12.2)		
	Traditional religion	2 (18.2)	9 (81.8)	11 (0.6)		
	No religion	9 (47.4)	10 (52.6)	19 (1.0)		
Wealth index	Poor	641 (56.1)	502 (43.9)	1143 (59.9)	0.36	0.834
	Middle	227 (56.0)	178 (44.0)	405 (21.2)		
	Rich	195 (54.3)	164 (45.7)	359 (18.8)		
BMI	Underweight	39 (55.7)	31 (44.3)	70 (3.7)	1.23	0.745
	Normal weight	612 (54.8)	504 (45.2)	1116 (58.5)		
	Overweight	287 (56.5)	221 (43.5)	508 (26.6)		
	Obesity	125 (58.7)	88 (41.3)	213 (11.2)		
Partner’s education level	No education	284 (56.7)	217 (43.3)	501 (26.3)	6.86	0.077
	Primary	176 (52.2)	161 (47.8)	337 (17.7)		
	Secondary	437 (54.5)	365 (45.5)	802 (42.1)		
	Higher	166 (62.2)	101 (37.8)	267 (14.0)		
Partner’s occupation	No Job	75 (59.5)	51 (40.5)	126 (6.6)	26.32	P < 0.01
	Public or private sector	152 (67.3)	74 (32.7)	226 (11.9)		
	Sales & services	120 (56.3)	93 (43.7)	213 (11.2)		
	Agricultural Sector	519 (56.3)	403 (43.7)	922 (48.3)		
	Skilled & Unskilled manual	186 (46.9)	211 (53.1)	397 (20.8)		
	Other	11 (47.8)	12 (52.2)	23 (1.2)		
Respondent’s occupation	No work	247 (57.8)	180 (42.2)	427 (22.4)	8.45	0.133
	Public or private sector	28 (63.6)	16 (36.4)	44 (2.3)		
	Sales & services	303 (52.0)	280 (48.0)	583 (30.6)		
	Agricultural Sector	470 (57.0)	354 (43.0)	824 (43.2)		
	Skilled & Unskilled manual	15 (58.3)	14 (41.7)	29 (1.6)		
Partner’s age	21–30	206 (47.8)	214 (52.2)	410 (22.9)	35.09	P < 0.01
	31–40	373 (53.5)	324 (46.5)	697 (36.5)		
	41–50	310 (60.1)	206 (39.9)	516 (27.1)		
	51–60	126 (66.7)	63 (33.3)	189 (9.9)		
	61–70	38 (70.4)	16 (29.6)	54 (2.8)		
	> 70	10 (80.0)	4 (20.0)	14 (0.8)		
Lack of Independency	No	325 (81.7)	73 (18.3)	398 (20.9)	136.94	P < 0.01
	Yes	738 (48.9)	771 (51.1)	1509 (79.1)		
Victims of emotional violence	No	899 (78.2)	251 (21.8)	1150 (60.3)	590.89	P < 0.01
	Yes	164 (21.7)	593 (78.3)	757 (39.7)		
Partner’s drinking habit	No	756 (65.3)	402 (34.7)	1158 (60.7)	108.84	P < 0.01
	Yes	307 (41.0)	442 (59.0)	749 (39.3)		

We want to measure the accuracy, precision, recall, and F1-score of seven machine learning algorithms on our data set. The prediction accuracy of the ANN, KNN, RF, DT, XGBoost, LightGBM, and CatBoost algorithms for our data set was 81%, 78%, 81%, 82%, 81%, and 82%, respectively, as shown in Table 9. In this instance, the DT and CatBoost algorithms produced the highest accuracy, precision, recall, and F1-score results. However, the KNN method had the lowest accuracy, precision, recall, and F1-score values. Overall, the DT method performed better than the other six algorithms for our prediction data set.

**Table 9:** Evaluation of the model’s performance

Models	Accuracy	Precision	Recall	F1 score
ANN	0.81	0.82	0.81	0.81
KNN	0.78	0.78	0.78	0.78
RF	0.81	0.81	0.81	0.81
DT	0.82	0.82	0.82	0.82
XGBoost	0.81	0.81	0.81	0.81
LightGBM	0.81	0.81	0.81	0.81
CatBoost	0.82	0.82	0.82	0.81

**Fig. 4:**
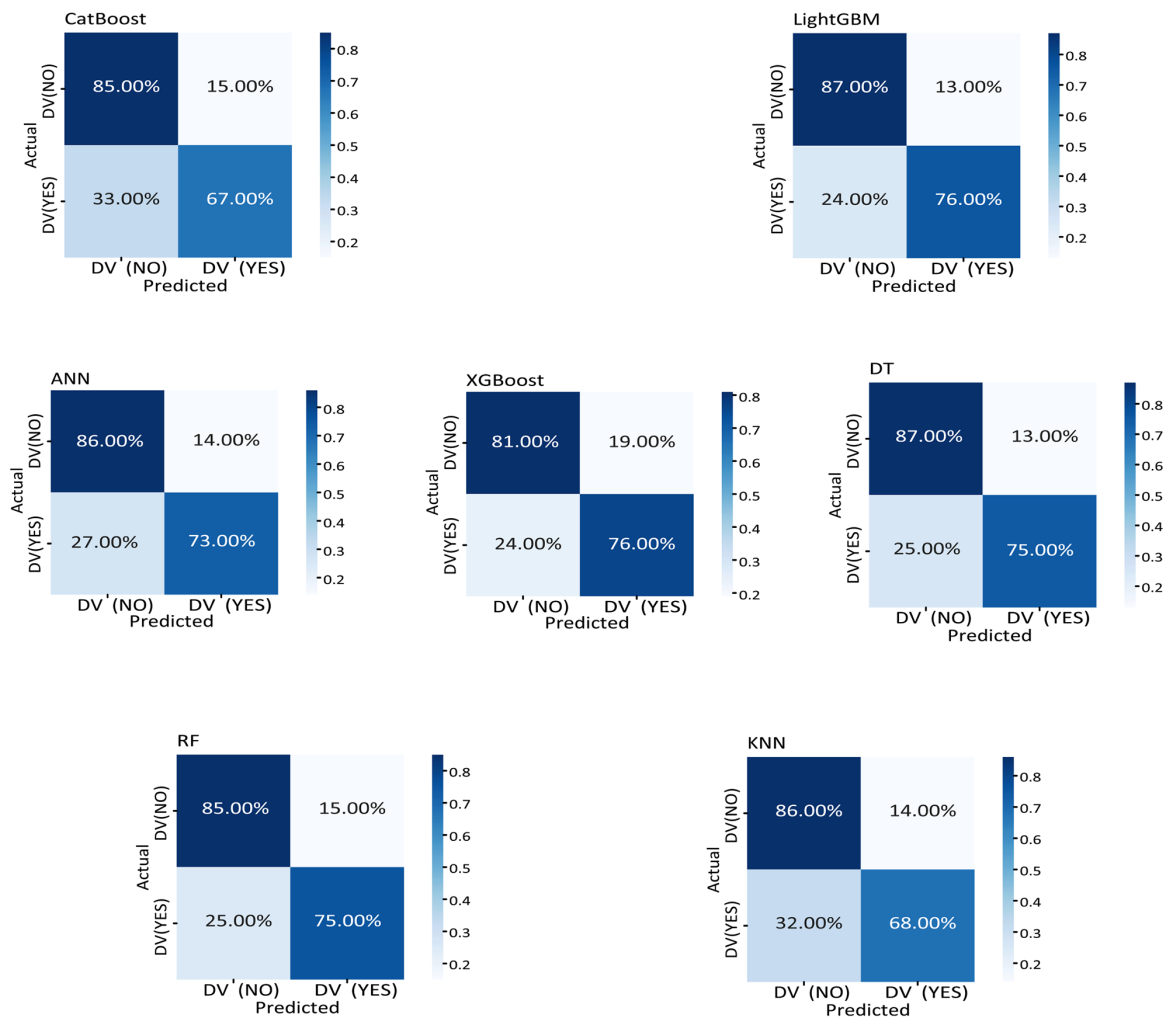
Confusion matrix of the ML models

Figure 4 shows the normalized confusion matrix for each of the following algorithms: ANN, KNN, RF, DT, XGBoost, LightGBM, and CatBoost. Figure 4 shows a confusion matrix that can help make this prediction result clearer. Figure 4 shows that the percentage true positive value in the confusion matrix of the LightGBM model is 76%. This means that it labels 76% of true positive values as true positive values. On the other hand, the number of true negative scores is 87%, which means that this model labels true negative classes as true negative 87% of the time. LightGBM gives the highest true positive value compared to other algorithms.

**Fig. 5:**
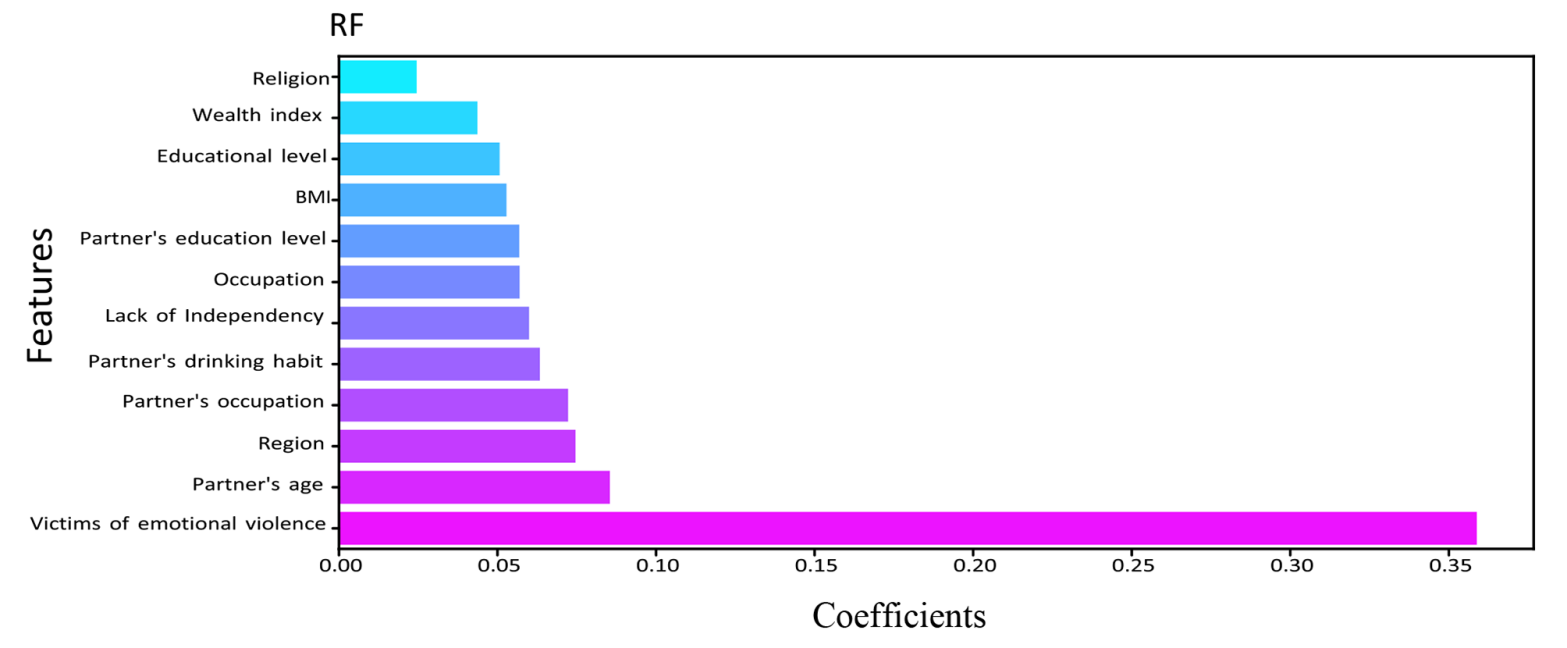
Feature importance of Random Forest (RF) model

**Fig. 6:**
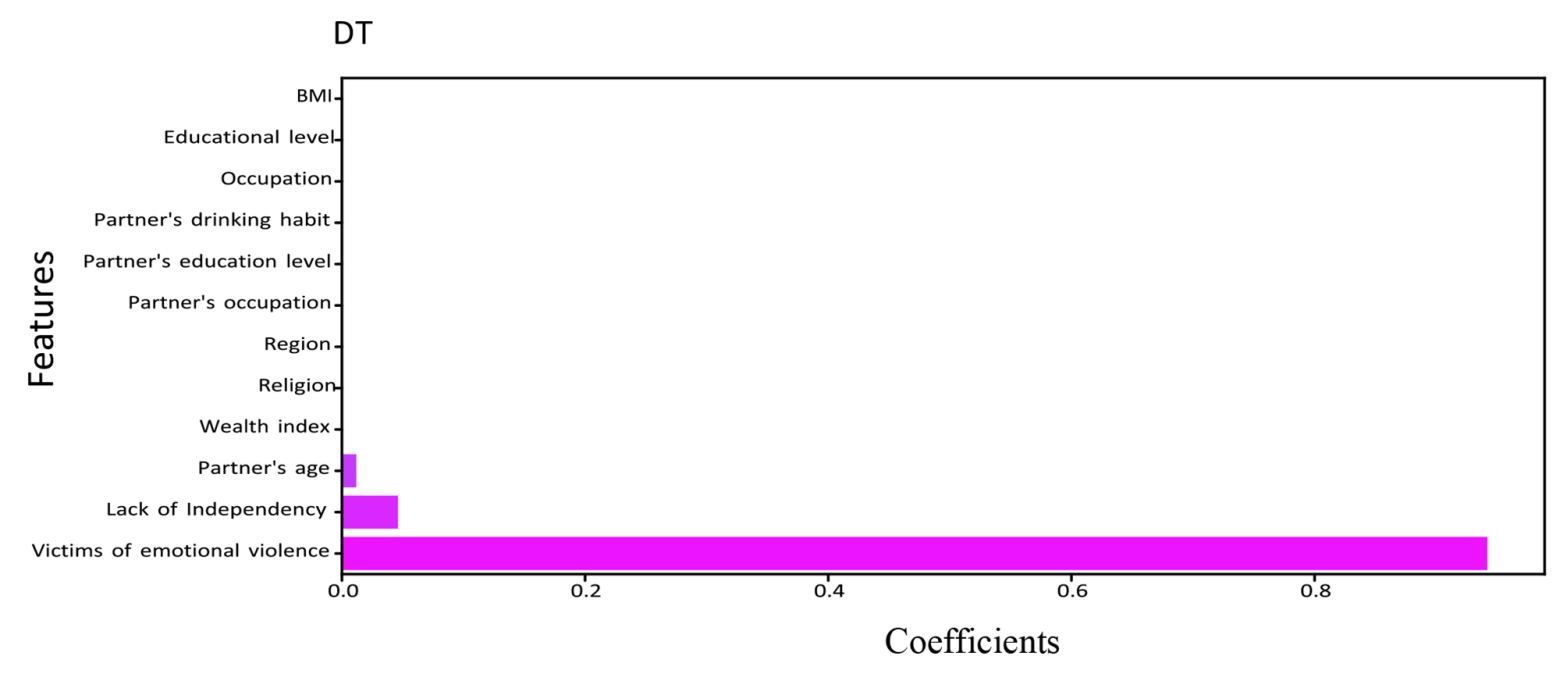
Feature importance of Decision Tree (DT) model

**Fig. 7:**
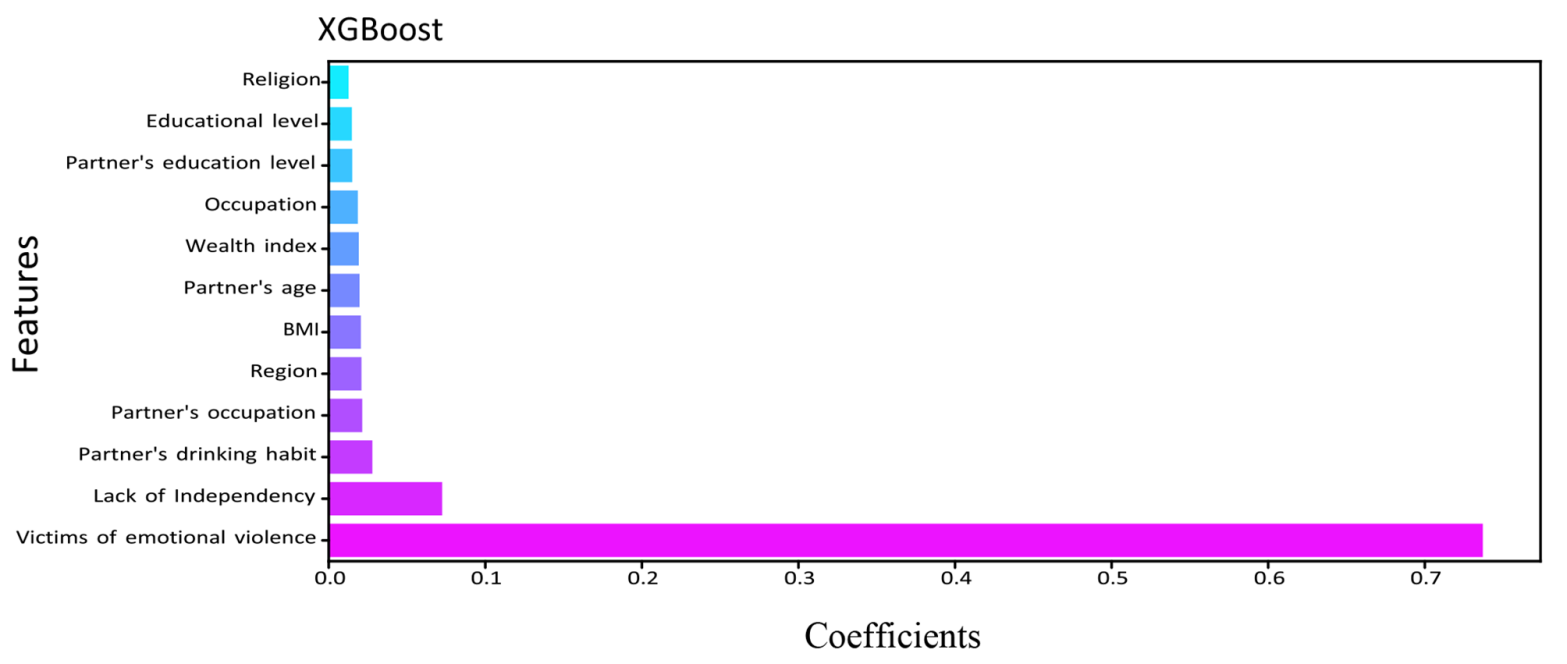
Feature importance of XGBoost model

**Fig. 8:**
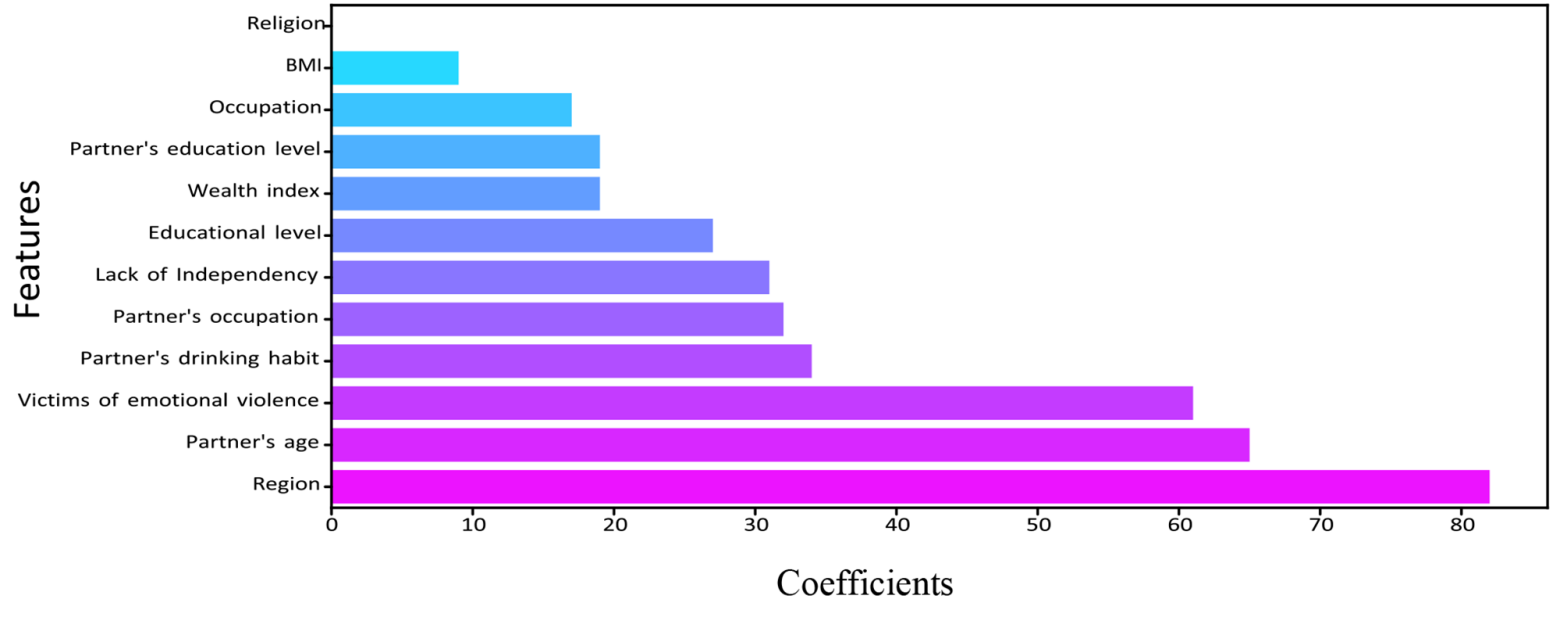
Feature importance of LightGBM model

**Fig. 9:**
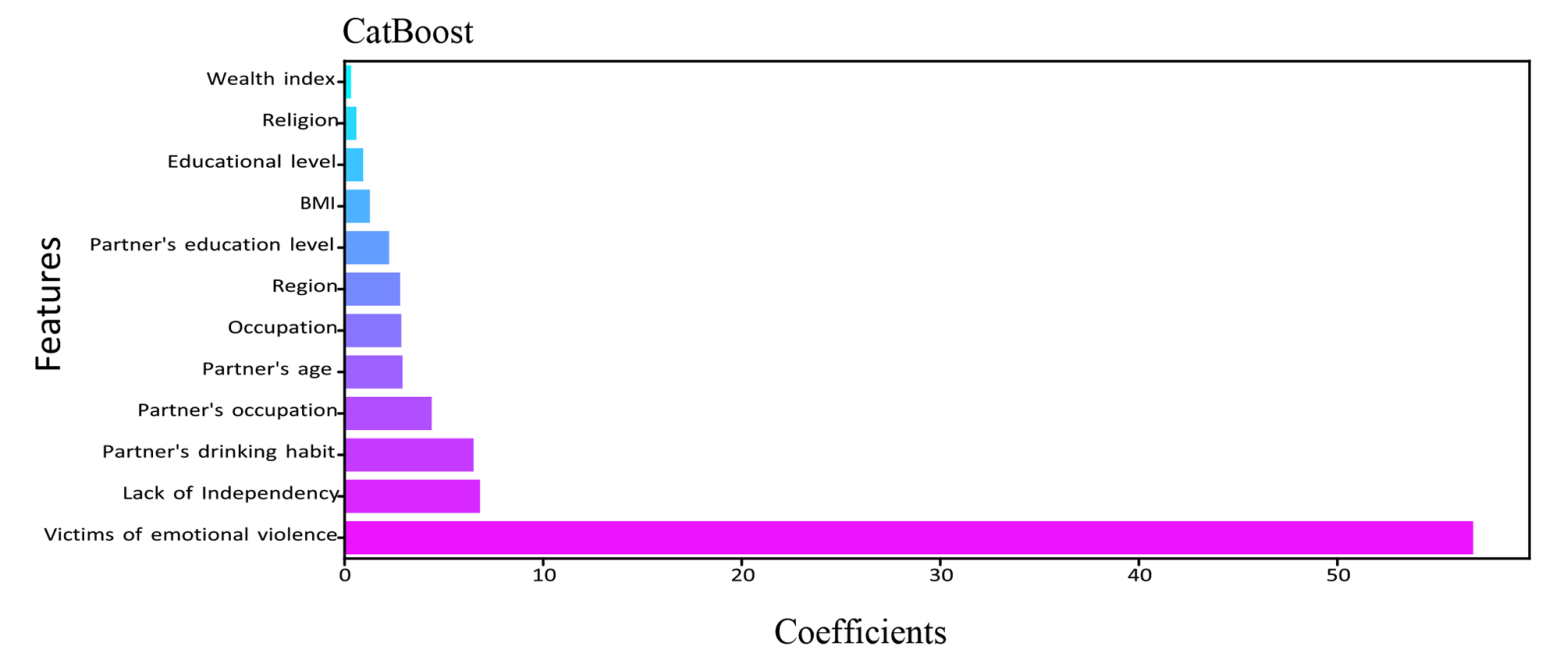
Feature importance of CatBoost model

The relevance of a feature describes whether certain data elements are more practical or significant than others. It can help to comprehend the solution better, and feature selection can occasionally result in model enhancement when the selected feature is implemented [46]. Feature importance is essentially a strategy that allocates a score to input features based on utility. They are adept at predicting a variable of interest. We determined the feature’s importance for RF, DT, XGBoost, LightGBM, and CatBoost. Figures 5, 6, 7, 8 and 9 illustrate the significant features of selected algorithms independently.

### RF

**Fig. 10:**
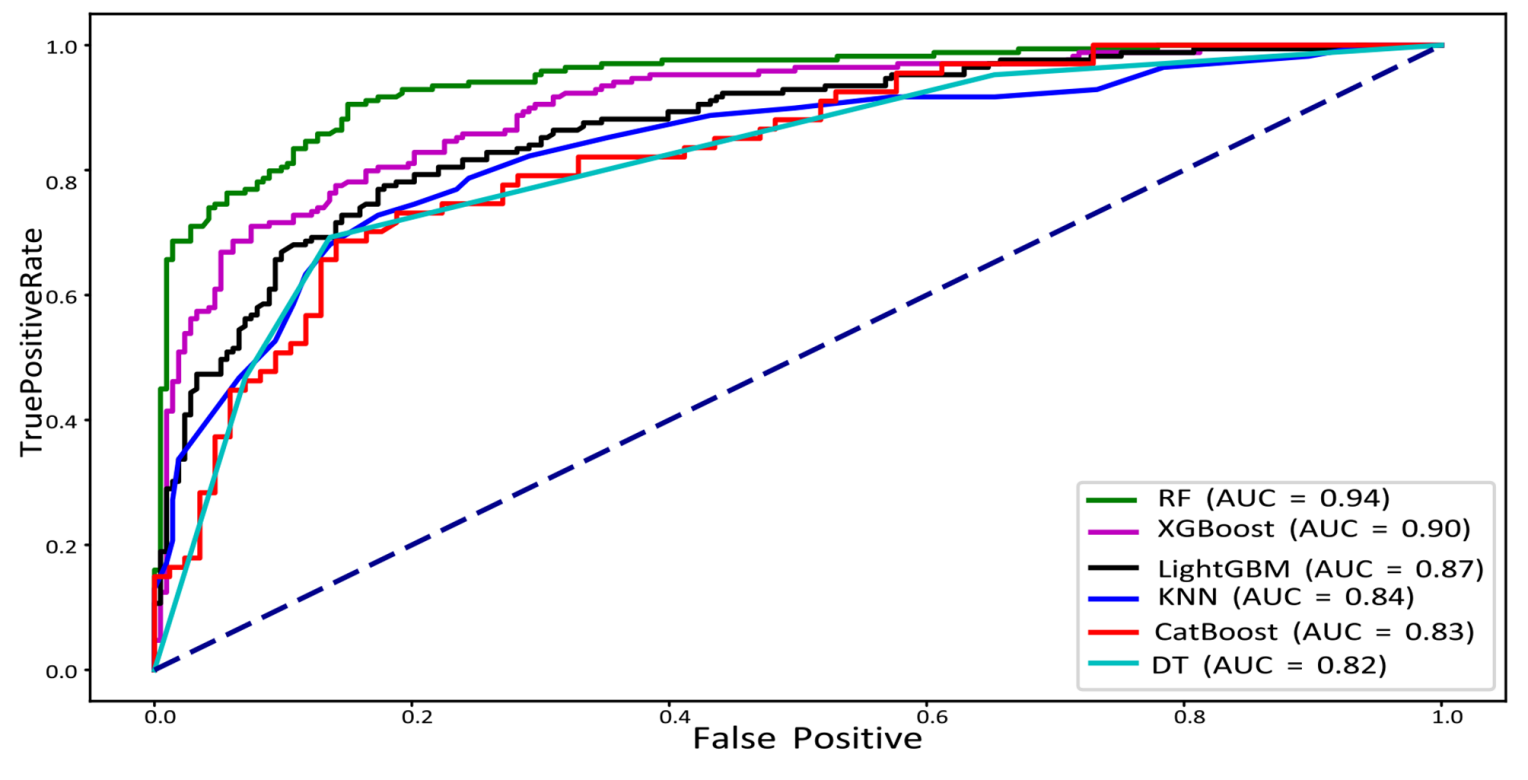
ROC curve of the ML models

The receiver operating characteristic curve, or ROC curve, is a graph that shows how well binary classifiers can diagnose problems. This study’s main goal was to find domestic violence cases and evaluate the results using seven different algorithms. Figure 10 demonstrates that the RF method has the best performance compared to the other machine learning algorithms.

## Discussion

Domestic violence is a serious issue affecting millions of women worldwide. In recent years, machine learning (ML) techniques have been increasingly utilized to identify and predict contributing factors to domestic violence. Our study is one of the major types of research that has used ML to predict domestic violence in Liberia. Previous studies by Amusa et al. (2022) [47], Petering et al. (2018) [48], and others have also employed ML strategies to predict domestic violence [49, 50].

Deep learning (DL) models, notably RNNs, have been the most popular computational strategy in ML in recent years, but they require vast amounts of data to function well [52]. DL also uses many layers of algorithms, each of which interprets data differently [51]. Our investigation was limited by lacking a big dataset for Liberian women’s domestic violence vulnerability prediction. While, traditional ML models are less likely to overfit, making them more resilient and effective with smaller datasets. Additionally, ML models like random forests (RF), decision trees (DT), and k-nearest neighbors (KNN) are easier to read and reveal feature importance. Interpretability is essential for research transparency and policymaker and stakeholder action for domestic violence prediction. Therefore, we used ML classifiers to analyze nationally representative survey data in Liberia. The model identified six factors that were significant among thirteen variables: educational level, partner’s employment, partner’s age, lack of independence, victims of emotional abuse, and partner’s drinking habit. This allowed us to automate the process of domestic violence prevention without requiring significant human effort.

Our findings reveal that 55.74% of Liberians have been victims of domestic violence at some point in their lives. However, women may be unable to take essential steps to address this situation. According to the World Health Organization (WHO), more than 50% of women in Bangladesh, Ethiopia, Peru, and Tanzania are highly exposed to domestic violence, which reaches an alarming 71% in Ethiopia [52]. Additionally, in South Africa and India, the respective percentages of domestic violence are 21.9% and 45.37% [47, 53].

In our investigation, most algorithms without KNN achieved the highest accuracy and recall ratings, ranging between 81% and 82%. In particular, ANN, DT, and CatBoost demonstrated the best accuracy compared to other research done in Bangladesh, while the RF model was the most accurate [47, 54]. Another study also implemented the RF model to predict domestic violence due to its ease of handling predicting errors and the provision of instructive visualizations [55, 56].

Our study identified several significant risk factors for domestic violence. Education level was a good predictor of domestic violence, consistent with previous studies [11, 54, 57]. In particular, illiterate women may be unaware of their rights, leading to a greater reliance on their partners and an increased likelihood of domestic abuse. Our study also revealed the partner’s drinking habit to be a risk factor for domestic violence, as the spouse may become disoriented due to alcohol consumption. A study conducted in Pakistan similarly found drinking to be a risk factor for domestic violence [10].

Furthermore, our study found that the partner’s age and occupation are also significant factors associated with domestic violence. A study conducted in Uganda supports these findings [58]. Women’s independence is a crucial factor in gender equality, which is one of the primary aims of sustainable development. Our findings suggest that a lack of independence is one of the most significant determinants of domestic violence, indicating that gender equality has not yet been achieved [59].

### Limitations

Our study utilized the LDHS 2019 dataset, known for its authenticity and adherence to DHS program requirements. However, since the survey was conducted a few years ago, the current values may have changed, which is a potential limitation. Additionally, since the study is cross-sectional, causal inferences and temporal trends could not be analyzed. Moreover, the analysis was limited to a specific set of variables due to the unavailability of DHS datasets. Nonetheless, our study provides valuable insights into the domestic violence features using machine learning algorithms, which have not been explored in depth before.

## Conclusion

In conclusion, our study utilized machine learning techniques to identify and predict contributing variables to domestic violence in Liberia. The results demonstrated that machine learning classifiers, such as CatBoost and DT, performed well in predicting domestic violence. However, there were some inconsistencies in the findings, indicating that researchers should consider hybrid machine learning approaches for superior results. We suggest that future studies combine data from DHS to enhance the efficacy of machine learning models. We also recognize the challenges of tuning hyper parameters to produce accurate results and determining relevant features. We recommend that policymakers and the government focus on the characteristics identified in our study, such as education level, partner’s employment, partner’s age, lack of independence, victims of emotional abuse, and partner’s drinking habit, to prevent domestic violence and promote gender equality.

## Data Availability

The data that support the findings of this study are openly available in The DHS Program at https://dhsprogram.com/data/available-datasets.cfm, reference country Liberia. All essential source code for this study will be accessible on Kaggle at the following link : https://www.kaggle.com/code/tingku/predicting-domestic-violence-in-liberia.
